# Sex Differences in Nutrition, Growth, and Metabolism in Preterm Infants

**DOI:** 10.3389/fped.2019.00022

**Published:** 2019-02-07

**Authors:** Pradeep Alur

**Affiliations:** Division of Neonatology, Department of Pediatrics, University of Mississippi Medical Center, Jackson, MS, United States

**Keywords:** sex, gender, infant, newborn, neonate, protein, growth, nutrition

## Abstract

Biological differences between the sexes are apparent even from the early part of the pregnancy. The crown-rump length is larger in male fetuses compared to females in the first trimester. Placentae of male and female fetuses have different protein and gene expressions, especially in adverse conditions. Even within the intrauterine milieu, the same extracellular micro RNA may show upregulation in females and downregulation in male fetuses. There appears to be a natural survival advantage for females. Maternal glucocorticoids (GC) play an important role in fetal growth and organ maturation. However, excess glucocorticoids can not only affect growth but the response may be sex-specific and probably mediated through glucocorticoid receptors (GR) in the placenta. Mild pre-eclampsia and asthma are associated with normal growth pattern in males, but in female fetuses, they are associated with a slowing of growth rate without causing IUGR probably as an adaptive response for future adverse events. Thus, female fetuses survive while male fetuses exhibit IUGR, preterm delivery and even death in the face of another adverse event. It is thought that the maternal diet may not influence growth but may influence the programming for adult disease. There is growing evidence that maternal pre-pregnancy overweight or obesity status is directly associated with a higher risk of obesity in a male child, but not in a female child, at 1 year of age. It is observed that exposure to gestational diabetes is a risk factor for childhood overweight in boys but not in girls. It is fascinating that male and female fetuses respond differently to the same intrauterine environment, and this suggests a fundamental biological variation most likely at the cellular and molecular level.

## Introduction

Sex differences in birth outcomes such as birth weights, and mortality were first reported by Clarke, in the Philosophical Transactions of the Royal Society in London in the year 1786 (1) He noted higher stillbirths and neonatal mortality amongst males than in females. He also observed a higher birthweight in male infants than in female infants (1). The biological differences between the sexes, however, become evident from the early part of the pregnancy. Premature births are reported to be more common in pregnancies with male fetuses. A 7.2% excess of males was found among white singleton preterm births (2). There is a 30% increased risk of spontaneous abortions for male fetuses with normal chromosomes (3). Male sex is consistently shown to be an independent risk factor for adverse pregnancy outcomes. In the early 1980s, researchers had demonstrated that fetal pulmonary maturity was higher in females and that androgens may inhibit surfactant production (4, 5). Evidence shows that females have an advantage over males, with a better outcome in the perinatal period, particularly after preterm birth (6). There appears to be a survival advantage for females, which is supported by the fact that under very harsh conditions, such as famines and epidemics, females live longer than men (7). In the famine in Holland brought on by military blockade during the World War II, male births decreased significantly more than female births suggesting a higher *in-utero* loss in males (8). The biological mechanisms involved in these sex differences remain to be explored. Since growth, nutrition and body metabolism are fundamental biologic processes, a comprehensive review of the literature on this subject may provide essential clues.

This review is organized to first review the sex differences in growth from conception through fetal and postnatal periods to early and late childhood. The second part of this review deals with differences in nutritional needs and metabolism.

## Sex Differences in Growth

Multiple factors influence fetal growth in human pregnancy. Much of the evidence comes from animal studies. There are not only fetal sex differences in growth; the sex-specific placental, hormonal, maternal anthropometric influences, and several yet unknown factors also appear to interact in many complex ways affecting fetal growth. The scientific evidence available so far only demonstrates the complexity of nature and does not provide conclusive evidence. As this is beyond the scope of this review to discuss all the available evidence, we will focus on the areas that have more significant clinical data.

### Growth Differences During the Fetal Period

Sex-specific growth differences in the fetus emerge quite early in the pregnancy. Initial studies could not conclude if the differences manifest in the first or second trimester (9, 10). Animal data, however, showed higher cell numbers in male embryos by 3.5 days in mice (11), and bovine male embryos were at an advanced stage of development compared to female embryos during the first 8 days (12). At least one human study showed that crown-rump length and biparietal diameter (BPD) in human male fetuses were on average larger than female at the first measurement between the 8th to 12th week (13). A recent study, however, suggested that small but consistent sex-related differences in prenatal BPD, head and abdominal circumferences measurements (higher in male fetuses) were established by as early as 15 weeks of gestation (14). Moore described significant differences in head growth trajectories between male and female fetuses. He further suggested that gestational age dating in the second trimester can be inaccurate if the BPD measurements are not sex-specific (15). Recently, the “Generation R” study of 1,782 pregnant women (a prospective population-based cohort study from fetal life until adulthood) concluded that crown-rump length was significantly larger in males compared to females in the first trimester (16). This study also noted that the head and abdominal circumferences were higher in male fetuses starting in the second trimester (16). Thus the growth of the male fetuses appears to be greater than the female fetuses from very early stages of gestation.

### Sexual Dimorphism in Placental Function vs. Fetal Sex in Growth

It is possible that differences in placental function might influence fetal growth and fetal programming in a sex-specific manner or fetal sex may determine placental function. Fetal sex-specific placental biomarkers were noted to be higher such as the pro-angiogenic placental growth factor (PlGF) and the anti-angiogenic soluble fms-like tyrosine kinase 1 (s-Flt1) in the first trimester in pregnancies with female fetuses (16). As the sex-specific growth differences seem to become evident starting in the first trimester of human pregnancy, maternal nutrients are unlikely to have any role to play at least until 11 weeks of pregnancy. There is evidence from embryological studies that early in the course of formation of the placenta, cytotrophoblast plugs obliterate the tips of the uteroplacental arteries preventing blood flow, as such fetal growth is unlikely to be dependent on hemotrophic nutrition in the first trimester (13).

In another study, maternal fasting glucose was significantly associated with placental weight in female fetuses but not in males (17). One of the hypotheses is that the sexually dimorphic differences in growth and survival of the fetus are mediated by the sex-specific function of the human placenta (18). In a baboon model of moderate maternal undernutrition, leading to IUGR, the male IUGR fetuses but not female IUGR fetuses, showed left ventricular fibrosis which inversely correlated with birth weight (19).

Maternal glucocorticoids (GC) play an important role in fetal growth and organ maturation. However, excess glucocorticoids can not only affect the growth but may also be sex-specific, and this is probably mediated through glucocorticoid receptors (GR) in the placenta. Excess maternal GC have shown to lead to reduced placental capillary length exclusively in male fetuses (20). Others have shown that GC may preferentially increase the production of reactive oxygen species in the placentas of male fetuses (21, 22). Animal studies have shown that synthetic GC may significantly reduce the expression of genes associated with fetal growth and nutrient delivery only in male and not in female fetuses (23). Moreover, evidence suggests that the placentae in female fetuses inactivate maternal GC more efficiently compared to males, through the action of placental 11 beta-HSD2. A decreased activity of this enzyme in placenta occurs in male fetuses and is associated with higher fetal cortisol. This higher intrinsic exposure to GC *in utero* may explain why male fetuses have poorer reserves compared to females with any maternal stress associated complications (24). High GC concentrations during sensitive windows in development could inhibit fetal cell division and differentiation, resulting in reduced fetal brain development and intrauterine fetal growth (25). It is recognized that male fetuses are resistant to excess glucocorticoids, whereas, female fetuses are highly sensitive (26). This finding is even more significant when repeated doses of GC in the form of maternal betamethasone (given to accelerate fetal lung maturation) may induce not only placental apoptosis (26) but also affect fetal growth by decreased circulating IGF and IGF binding proteins leading to reduced fetal growth (27).

The effect of GC on fetal and placental tissue may also be mediated via the glucocorticoid receptor (GR). There are eight isoforms of GR in human placenta. Preterm small for gestation (SGA) female placentas have decreased expression of nuclear GR-A and GRa-D2 receptors. The GRa-D2 receptor is associated with decreased transcription of glucocorticoids and may be associated with heightened inflammation as this GR isoform is less sensitive to glucocorticoids and their anti-inflammatory effects (28). GRa-C is the most potent activator of glucocorticoid-induced apoptosis, and it is enhanced in both male and female preterm placentae, and thus may be activated before the preterm onset of labor (29). The effect on GR may be related to isoform differences or due to epigenetic modifications. Recently, an *in-vitro* study showed that differential DNA methylation of GR is associated with large for gestation status at birth (30). This further supports that GC and differential actions mediated through GR affect the fetal growth significantly. A hypothesis was proposed to summarize the interactions between maternal nutrition, GC and GR. The stress of maternal undernutrition increases maternal GC with a reduction in GR, and a decreased expression of placental 11 beta HSD-2. The 11 beta HSD-2 enzyme is considered as an anti-obesogenic, and its decreased activity is associated with increased fetal GC and GR. On the other hand, there is increased expression of a related enzyme, 11beta-HSD1in the fetal adipocytes, which re-amplifies local tissue GC and thus predisposing to fetal lipogenesis which is a precursor for adult adiposity (31).

Placental transporter voltage-dependent anion-selective channel protein 1, a major calcium transport channel critical for fetal development is preferentially enhanced in male placentas. This preferentially enhanced activity may predispose male fetuses to toxicants that can hijack this receptor during prenatal life (32). It seems that the placentae of male and female fetuses have different protein and gene expressions, especially in adverse conditions. Males usually respond with minimal gene and protein changes in the placenta with continued growth in a sub-optimal intrauterine environment, which puts them at high risk for IUGR, preterm birth or even death if another acute adverse event occurs (18). The females, in contrast, express multiple placental genes and protein changes that result in a milder decrease in growth without actual growth restriction (>10th centile) (18). These female adaptations in placental function and growth seem to enable them to be better prepared for another adverse event, which may further compromise nutrient or oxygen supply (18). These fetal observations afford support to the anthropological observations that females survive adversities better than males.

### Sex-Specific Biomarkers During Pregnancy

A large prospective study of placental biomarkers in complicated and normal pregnancies, showed that sex-specific placental biomarkers become evident in the first trimester itself (33). During the first-trimester certain placental and vascular growth related molecules such as soluble Fms-like tyrosine kinase (s-Flt1), placental growth factor (PLGF), plasminogen activator inhibitor (PAI-2), are higher in placentae of female fetuses as compared to male fetuses. The sex-specific differences, however, disappeared once the vascular complications such as pre-eclampsia or growth restriction supervened (33).

Sex differences are noted even at the level of microRNA expression. miRNAs constitute a highly conserved class of small non-coding RNAs, involved in post-transcriptional regulation processes by modifying the expression of specific mRNAs. Specific miRNAs regulate the various aspects of placental development such as cell differentiation, adhesion, migration, apoptosis, and angiogenesis. Aberrant expression has been associated with the pathogenesis of pregnancy-related complications (34). A recent study which looked at the extracellular micro RNAs (miRNA) during the second trimester found a correlation between fetal sex and fetal growth. One study compared the IGF2-derived intronic miR-483-3p in the macrosomic but non-diabetic placentas and normal pregnancy placentas. It noted that miR-483-3p was overexpressed in macrosomic placentas. As miRNAs play an important role in the development of the fetus and the placenta by regulating their target genes, their overexpression is thought to contribute to the placental cell proliferation and macrosomia as a consequence (35). Sex-specific differences in the levels of miRNAs were noted when comparing mothers of large-for-gestational-age (LGA) vs. appropriate-for-gestational-age (AGA), but not in small-for-gestational-age (SGA) vs. AGA infants (36). Most of the miRNA were upregulated in females and downregulated in males (36). Further studies may be able to delineate if these markers can be used to monitor fetal growth during the second trimester. Another study from the same group found that fetal sex regulated the expression of miR-210 in the placenta via the NFkB1 pathway and estrogen levels in obese pregnant women, a condition known to be a risk factor for LGA pregnancies (37). Sexual dimorphism in fetal cardiac miRNAs under conditions of intrauterine growth restriction has also been linked to the NFkB1 pathway (16). miRNAs are currently of great research interest due to their possible role in the antenatal diagnosis of trisomies, and association with complications of pregnancy, in addition to their role in fetal growth. However, the evidence is in the very early stages of discovery.

Metabolism-associated genes such as Humanin, which increase insulin sensitivity and are linked with fetal growth are upregulated in males. Such preferential activation may give an insight into the higher growth rates seen in male fetuses (38).

### Sex-Specific Fetal Growth in Natural vs. Assisted Reproductive Pregnancies

The process of assisted reproduction introduces some potential stressors, including, *in-vitro* media, handling of embryos, temperature and light fluctuations, ICSI, and prolonged culture (20). These factors have the potential to affect the growth of the embryos and the fetuses. However, a recent study showed that sex-specific differences in growth persist with assisted reproductive therapies; and are not exaggerated (39).

### Sex-Specific Fetal Growth and Influence of Maternal Conditions

When pregnancy is complicated with mild asthma, female fetal growth was reduced but not to the extent of causing IUGR. In contrast, male fetuses grew normally unless complicated by acute asthma exacerbation, which led to IUGR status or preterm delivery (18). Mild pre-eclampsia was associated with normal growth trajectories of the male fetus and growth reduction in the female fetus (40). This again reiterates other observations that show how female fetuses curtail their rate of growth, perhaps as a survival strategy in preparation for multiple insults in the future.

### Sex-Specific Fetal Growth and the Influence of Maternal Anthropometry

Researchers have reported sex differences in fetal growth in relation to maternal height and weight. A male advantage of 60 g occurred among neonates of the shortest and lightest mothers (41). Fetal sex as an independent factor seems to influence placental weight with higher fetal: placental ratio in boys (17). Though studies have described a strong association of gestational weight gain with postnatal obesity (42, 43), only one recent study, involving 955 mother-infant pairs, has suggested that maternal pre-pregnancy overweight or obesity status was directly associated with higher risk of obesity in a male child at 1 year of age (44). In a study that included 9,270 consecutive women with singleton pregnancies and without a previous diagnosis of diabetes mellitus, it was noted that maternal BMI positively correlated with LGA and macrosomia in both male and female fetuses. However, a negative correlation between maternal BMI was seen with SGA births only in males (45). Hence, sex-specific interactions between maternal BMI and fetal weight need further larger studies to delineate the differences.

### Maternal Nutrition

It is interesting to speculate that maternal nutrition could influence fetal growth. However, the evidence shows an incomplete understanding of the mechanism at this point. It is believed that the fetus does not depend on the maternal diet especially in the first trimester, and probably in the second trimester as well, but depends on the stored maternal nutrients. It is thought that maternal diet may not influence growth but may influence the programming for adult disease. However, protein supplementation during pregnancy resulted in lower birth weights, and similarly, high protein to carbohydrate diet was associated with raised blood pressure in the offspring in adult life (46). Hence, a balanced diet, especially during pregnancy, may have long-term benefits. It is argued that the male fetus is adopting a more dangerous strategy that puts it at greater risk of becoming undernourished with preference to rapid body growth and brain growth rather than combining with placental growth. It is also proposed that boys are more responsive to the mother's current diet than girls, who respond more to their mother's lifetime nutrition and metabolism (47).

### Sexual Dimorphism in Outcomes of Aberrations in Fetal Growth

Research suggests that extremes of fetal growth may increase the susceptibility to adult diseases through cellular memory. Studies show that both intrauterine growth restriction, and excessive growth had epigenomic responses. Both patterns of growth were associated with DNA hypermethylation targeting cis-regulatory elements in proximity to genes involved in glucose homeostasis and stem cell function (48). This may explain how intrauterine growth restriction and overgrowth could both be associated with increased risk for type 2 diabetes as adults. Interestingly, the same researchers also noted sexually dimorphic responses in patterns of growth. Intrauterine growth restricted (IUGR) males and large for gestational age females had epigenetic dysregulation (48). Other reports show similar DNA hypermethylation in the promoter and enhancer regions of the genome in CD3 (+) T cells of IUGR male infants (49). These may be the biomarkers, which could potentially predict metabolic disorders when these infants grow into adulthood.

In the OBEGEST cohort study, which looked at the association between gestational diabetes (GDM) and child overweight at 5–7 years, it was observed that exposure to GDM is a risk factor for childhood overweight in boys but not in girls (50). It is fascinating that male and females respond differently to the same intrauterine environment, and this suggests a fundamental biological variation most likely at the cellular and molecular level.

### Growth and Sex of the Twin-Pairs

In a large observational study of same-sex twin pairs vs. mixed-sex twin pairs pregnancies, male-male twins had a higher risk of respiratory distress syndrome and lower incidence of being IUGR compared to female-female twin pairs (51). This suggests that apart from intrinsic differences within fetal sexes, the hormonal and environmental influences in twins may also be sex-specific. Though several hypotheses have been proposed to explain the above differences, there is no strong evidence yet available.

### Sexual Dimorphism in Fetal and Preterm Anthropometry

Insulin-like growth factor-1 (IGF-1), the main growth-promoting factor during intrauterine life is higher in females than in males along with higher levels of IGF binding protein-3 (IGFBP-3) (52, 53). Though its significance is unknown, IGF-1 levels correlate with short-term growth velocity and protein intake in preterm infants (54, 55). In a recent cross-sectional cohort analysis of 369 neonates for cord blood insulin, and insulin-like peptides, IGF-1 was closely associated with birth weight in males. Whereas, C-peptide correlated with female placental weight and birth weight. Though these results emphasize sex-specific differences, their biological significance is unknown. It is well-established that fat mass (FM) is higher and lean body mass (LBM) is lower in female children (56), however, these anthropometric differences appear to be present even in preterm infants. The Intergrowth-21st project showed that boys on an average had more fat-free mass than girls' at all gestational ages after 34 weeks of gestation (57). At birth, FM in boys was 9.9 vs. 11.0% in girls, and the difference persisted over time (57). Not only the fat mass but also the fat distribution in preterm infants is different between the sexes. Preterm and term females have a more centralized pattern as well as the larger amount of subcutaneous fat compared to males (58).

Others have observed that body weight, gender, and length can predict lean mass, fat mass and percentage fat mass accurately using DEXA scan at birth with significantly lower lean mass and higher fat mass and percentage fat mass in girls (59). Similarly, using air-displacement plethysmography, normal reference ranges for fat mass, the percentage of fat mass and fat-free mass (lean mass) based on gender and gestational age were created, which also showed similar sex-specific differences as DEXA scan (60). This study also showed that the percentage of fat mass increased from 36 to 40 weeks of gestation (60).

It appears that subcutaneous fat is affected by intrauterine growth unlike intra-abdominal fat, as revealed by magnetic resonance imaging, indicating these two fat regions are under different physiological influences (61). Skinfold thickness has been shown to correlate with total fat mass, and may thus be a simpler and more practical measurement for clinical use. Sex-specific total (from 4 areas in the body) skinfold thickness normative data have been published (62).

Research as far back as 1,923 described that the amount of adipose tissue explained the variability in weight within mammalian species whereas the amount of lean body mass was relatively constant and changed consistently over the lifetime (63). Overall, male newborns tend to have higher fat-free mass than females.

## Sex Differences in Nutrition and Metabolism

It is well-known that weight, length, and head circumferences are greater in male preterm infants at all gestational ages (64). It may be that cell division in male embryos occurs more rapidly than those in female embryos (65). We, therefore, have sex-specific growth charts such as Fenton-2013 for the preterm infants starting from 22 weeks of gestation (64). It is intuitive to extrapolate that, if growth rates are different between male and female ELBW infants, then their nutritional requirements would also be different. However, there is a lack of information on this subject.

### Preliminary Evidence

After failing to find sex-specific protein and caloric requirements recommendations in the literature, we published a preliminary report on this topic (66). We retrospectively reviewed ELBW infants born in our institution to study if male and female infants received differing amounts of mean daily protein, and calories while on full enteral nutrition to maintain appropriate for gestational age status for both weight (WT) and head circumference (HC) from birth to discharge (66). The protein and calories during enteral nutrition and morbidities between male and female ELBW infants that maintained appropriate for gestational age (AGA) status from birth to discharge were compared. To maintain AGA status for both HC & WT, the mean protein and calorie requirements for males were significantly lower than for females [Protein (g/kg/day): 3.53 vs. 3.71 (*P* = 0.003) and Calories (Cal/kg/day): 118.7 vs. 123.5 (*P* = 0.002)]. However, the discharge outcomes were not different between the sexes despite differences in protein and calorie provision during enteral feedings, suggesting that female ELBW infants may need higher protein and calories.

Though this study does not prove that sex differences exist in the nutritional requirements, it indeed generates the hypothesis that needs to be addressed by a larger prospective study. However, we recognize that it will be challenging to prospectively study the differences in nutritional requirements between the sexes focusing on aspects other than the SGA rates after providing standard calories and protein.

### Sex Differences in the Effect of Nutrients on the Postnatal Outcomes

A retrospective study from the Netherlands showed that increased amino acid and energy intakes during the first week of life were associated with a statistically significant short-term improvement in weight gain in male very low birth weight infants compared to females (67). Another study in preterm infants of < 32 weeks compared the administration of glucose alone, to glucose along with 2.4 g/kg/d of amino acids, in the first few days after birth. Neurodevelopmental follow-up at 2 years of age showed that male infants benefited more from early amino acid administration compared to female infants (68). They concluded that premature males, but not females, had a normal developmental outcome from amino acid administration immediately following birth.

One of the studies showed that even breast milk might have a differential effect on white matter growth in males vs. females (69). In this study, it was shown that there was a dose-response relationship between early breast milk intake in preterm infants of ≤ 30 weeks gestation and later IQ scores at 7–8 years of age, and this effect was more pronounced in males (69). Another large study demonstrated that male neurodevelopment is much more sensitive to early growth in the neonatal intensive care unit than female very preterm infants (70). Poindexter et al. in their “early vs. late amino acid initiation study” noted that males in the late amino acid administration group had increased odds of having a suboptimal head circumference at 18 months corrected gestational age; with the odds ratio of 3.3 (95% CI, 1.4 to 7.7) for males having a head circumference of less than the 5th percentile (71).

### Sex-and Maternal Glucose and Leptin Metabolism

#### Glucose Metabolism

Total glucose metabolism was noted to be twice as high in male bovine embryos compared with female bovine embryos (72). Recently, it was shown that fetal sex might influence maternal plasma glucose levels. The presence of male fetuses was independently associated with elevated maternal fasting plasma glucose and lower basal β-cell function (73, 74).

#### Role of Leptin

Cord blood levels of leptin have been correlated with newborn adiposity (75). Recently leptin levels in cord blood plasma were found to be lower in male term infants compared to female infants (76). Leptin levels correlated positively with birth weight. Since the fat mass percentage is higher in girls compared to boys, higher leptin levels in girls seem intuitive. However, it appears that maternal leptin does not correlate with newborn adiposity in either sex, maternal adiponectin levels positively associated with the fat mass percentage in male fetuses (77).

#### Thyroxine Maturation

**Figure 1:**
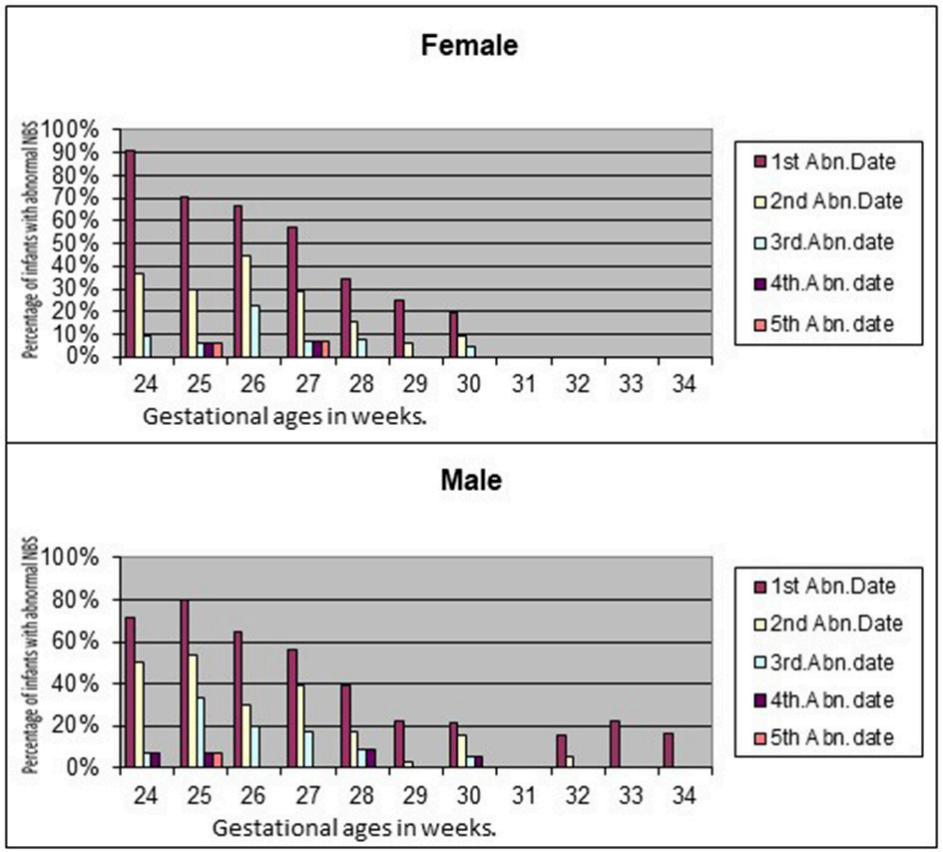
Sex differences in normalization of T4 in preterm infants.

There is scant literature on sex differences in thyroid maturation in preterm infants. We undertook a pilot study to understand the corrected gestational age at which low thyroxine (T4) levels in preterm infants normalize in the universal state newborn metabolic screening test (NBS) (57). Of the 355 infants included in the study, 127 had low T4 with normal TSH on first NBS. Mean adjusted gestational age at which all the study infants had normal results was 31.13 weeks. No infant developed high TSH in the repeat screenings. No female (0/53) between 31 and 34 weeks gestation had low T4 compared to 13.8% of male infants (6/45; *p* = 0.007) in the initial NBS. Female preterm infants normalized thyroxine levels earlier than males. None of the female infants had abnormal T4 levels after 30 weeks of gestation (Figure 1). Nonetheless, these observations need to be confirmed with larger studies.

## Conclusions

Sex differences in growth are obvious as early as the first trimester (Table 1). There is growing evidence of sexual dimorphism in genetic, environmental and epigenomic responses to intrauterine growth aberrations. Sex differences in anthropometry seem to become evident even in very preterm infants. Failure to use sex-specific BPD measurements may cause errors in dating during the second trimester. It is now clear that the growth of the male fetuses is greater than the female fetuses from very early stages of gestation. There appears to be a complex interaction between the placenta and fetal sex. The evidence is pointing toward the greater manifestation of placental biomarkers in female fetuses' placentas. Both the animal and human studies indicate that female fetuses may tolerate excess glucocorticoids better than the male fetuses. The female adaptations in placental function and growth seem to enable them to be better prepared for adverse events than the male fetuses. The micro-RNAs play an important role in the development of the fetus and the placenta by regulating their target genes; their overexpression is thought to contribute to the placental cell proliferation and macrosomia, therefore. It seems that most of the miRNAs are upregulated in females and downregulated in male fetuses in mothers with large for gestational age fetuses.

**Table 1:** Summary of sex-specific differences in growth and metabolism.

	**Male**	**Female**
**GROWTH-1ST TRIMESTER**		
Crown-rump length	Larger	Smaller
**GROWTH-2ND TRIMESTER**		
Biparietal diameter	Larger	Smaller
Abdominal circumference	Larger	Smaller
**GROWTH-3RD TRIMESTER**		
Fat mass	9.9% at birth	11% at birth
Lean body mass	Higher	Lower
**MATERNAL MORBIDITY**		
Mild Pre-eclampsia	Normal growthrate	Reduced growthrate
Maternal obesity	Higher risk of obesity at 1year	No definite effect
Gestational diabetes	High risk of beingoverweight at 5–7 years	No known risk of beingoverweight
**OTHER FACTORS**		
Placental gene expressions	Minimal gene and protein changes	Multiple gene and proteni changes
Same sex twin pairs	High risk for RDS and low risk for IUGR	High risk for IUGR
IGF-1& IGFBP-3 levels	Lower	Higher

The role of maternal diet in fetal growth is unclear still. It is proposed that boys are more responsive to the mother's current diet than girls, who respond more to their mother's lifetime nutrition and metabolism (47). In the OBEGEST cohort study it was observed that exposure to GDM is a risk factor for childhood overweight in boys but not in girls (50).

There are sex-specific growth charts, and growth outcomes are different between the sexes in preterm infants. The preliminary data about the sex-specific nutritional requirements in preterm infants warrants further research. Interestingly, early studies have shown that fetal sex might influence maternal plasma glucose levels.

Thus, the existing evidence points out natural sex differences in several placental biomarkers. However, their metabolic significance still needs to be established. We hope to see how these natural sex differences in very early in life can impact the long-term metabolism and disease onset in later life. Such information may give us clues to better management of pregnancy for healthy adult life.

## Author Contributions

The author confirms being the sole contributor of this work and has approved it for publication.

### Conflict of Interest Statement

The author declares that the research was conducted in the absence of any commercial or financial relationships that could be construed as a potential conflict of interest.
